# Transactional Sex among Noninjecting Illicit Drug Users: Implications for HIV Transmission

**DOI:** 10.1155/2016/4690628

**Published:** 2016-08-28

**Authors:** Rafael Alves Guimarães, Aurélio Goulart Rodovalho, Inaina Lara Fernandes, Graciele Cristina Silva, Rodrigo Lopes de Felipe, Ivânia Vera, Valéria Duarte Gregório, Roselma Lucchese

**Affiliations:** ^1^Federal University of Goiás, Regional Goiânia, Goiânia, GO, Brazil; ^2^Municipal Health Department of Catalão, Catalão, GO, Brazil; ^3^Federal University of Goiás, Regional Catalão, Catalão, GO, Brazil

## Abstract

Noninjecting illicit drug users (NIDUs) present high risk for HIV infection, due especially to transactional sex. This study aimed to estimate the prevalence and risk factors for transactional sex among NIDUs in the Southwest region of Goiás State, Central Brazil. The prevalence of self-reported transactional sex was 22.8%. Prevalence in women and men was 52.7% and 16.8%, respectively, a significant difference (*p* < 0.001). Crack use and history of sexually transmitted infections (STI) were risk factors for transactional sex in men. Homelessness, crack use, sex under the influence of drugs, and history of sexual violence were risk factors for transactional sex in women. A high prevalence of transactional sex was observed among NIDUs. This risk behavior may contribute to the high rates of HIV among this population and their social networks and in the general population.

## 1. Introduction

Noninjecting illicit drug users (NIDUs), particularly those taking crack, are very vulnerable to Human Immunodeficiency Virus (HIV) infection. They are particularly affected by HIV infection worldwide, primarily because of the elevated rates of high-risk behaviors, such as inconsistent condom use, multiple sexual partners, sex under the influence of alcohol, and/or drugs and transactional sex [1].

Transactional sex, defined as the exchange of sex for money, drugs, food, shelter, or other items [2], is a way for drug users to earn an income to finance their high drug consumption. Studies have shown high rates of transactional sex among NIDUs, and this behavior is strongly associated with HIV infection [1, 3–6] in these populations. In this respect, transactional sex among NIDUs increases the risk of spreading HIV to their social networks and the general population. Some factors, such as female sex, crack use, mental illness, homelessness, binge drinking, sexual violence, and risk behaviors [6, 7], have been associated with transactional sex among illicit drug users. Knowledge of these factors can contribute to public actions and policies to prevent and control STI in NIDUs, such as intensifying health promotion activities for certain subgroups that are more vulnerable to transactional sex.

Studies have focused on the sexual behavior of injecting drug users. Accordingly, research on transactional sex rates and patterns among NIDUs is scarce, especially in countries with high noninjecting drug consumption rates, such as Brazil. This study aimed to estimate the prevalence and risk factors for transactional sex among NIDUs in the Southwest region of Goiás state, Central Brazil.

## 2. Methods

A cross-sectional study was carried out from October 2014 to June 2015 with NIDUs from two drug rehabilitation treatment centers in Goiás state, Central Brazil. Clinics participating in this study provide free treatment for individuals diagnosed with mental and behavioral disorders due to the use of psychoactive substances (alcohol and injecting and noninjecting illicit drugs), through the Brazilian National Health System or health insurance. The institutions work full-time, assisting outpatients and intensive care patients, with voluntary admissions.

The inclusion criteria were patients (1) aged 18 years or over; (2) undergoing treatment with medical diagnosis of mental and behavioral disorders due to the use of psychoactive substances (except alcohol and tobacco), according to ICD-10 [8], previously confirmed from medical records, and (3) having consumed noninjecting drugs in the 30 days before admission (marijuana, intranasal cocaine, crack, inhalants, d-lysergic acid diethylamide [LSD], and ecstasy), according to self-reports. Individuals who were in an apparent state of excitement or sedation prior to the interview and who used intravenous drugs in their lifetime were excluded.

Subjects were recruited from the clinics, in the morning or evening, by health experts and members of the study team. Based on a list provided by the directors of the institutions, all potentially eligible individuals were invited to participate in the research during the data collection period. After giving their informed consent, all patients were interviewed by a team of healthcare professionals previously trained in conducting interviews and in the research methods. Individuals took part on a voluntary basis and received no remuneration.

The study questionnaire was based on previous studies with vulnerable populations and included sociodemographic variables, use of alcohol and drugs, transactional sex, and other risk behaviors for STI. A team of specialists in sexual behavior and mental health evaluated the questionnaire, which was applied as a previous pilot test to a sample of NIDUs with similar sociodemographic profiles.

Transactional sex in the last year, the primary variable of interest in this study, was derived from the following question: “*Have you received money and/or drugs in exchange for sex in the last year?”* The definition adopted in this study was money or drugs obtained consensually, excluding nonconsensual transactional sex and based on previously conducted studies [2, 6, 9].

The following independent variables were analyzed: age (years); crack use (yes or no), intranasal cocaine use (yes or no), marijuana use (yes or no or), LSD use (yes or no), ecstasy use (yes or no), inhalant use (yes or no), tobacco use (yes or no), binge drinking (yes or no), being homeless in the previous six months (yes or no), history of sexual violence by any stable or casual partner in the previous year (yes or no), history of STI in the previous year (yes or no), condom use with any casual sexual partner in the previous year (always or sometimes/never), sex under the influence of alcohol (yes or no), sex under the influence of illicit drugs (yes or no), and sex with injecting drug users in the previous year (yes or no).

Binge drinking was defined as the consumption of five or more shots of alcoholic beverages on a single occasion for men and four or more for women. Sexual violence was assessed by the question: “*In the last year, did anyone force you to have sex against your will*?” History of STI was determined using the following question: “*Have you been diagnosed with an STI/venereal disease in the last year*?” Information regarding sex under the influence of alcohol, illicit drugs, and sex with illicit drug users was obtained by the questions: “*Have you used alcohol before or during sex in the last year*?” “*Have you used illegal drugs before or during sex in the last year*?” “*Have you had sex with injecting drug users in the last year*?”

Data were analyzed using STATA 12.0 software. Descriptive statistics were used to present sociodemographic and behavioral characteristics. Age and family income were presented as median and interquartile range (IQR) and categorical variables as relative frequency. Prevalence of transactional sex was estimated using 95% confidence intervals (95% CI). Bivariate analysis was performed between the outcome and potential risk factors. Subsequently, variables with *p* < 0.10, derived from bivariate analysis, were included in the Poisson regression models [10]. In this investigation, due to prevalence differences between the sexes, risk factors were analyzed separately for men and women.* p* values <0.05 were considered statistically significant.

This study was approved by the Federal University of Goiás Research Ethics Committee, under protocol number 926819/2014. Consent was obtained from all participants.

## 3. Results

During the study period, 380 individuals were invited to participate. Of these, 15 refused, 20 were in an apparent state of sedation and/or agitation before the interview, and 22 had injected illicit drugs during their lifetime, according to self-reports. Thus, 323 NIDUs were recruited, representing 85.0% of individuals admitted to the institutions, in the morning and afternoon.

Most participants were men (83.0%), single (72.1%), and self-reported as brown or dark-skinned (59.1%). The median age was 32.0 years (IQR: 15) and the median monthly family income USD 625.68 (IQR: 762.82). Users reported consumption of crack (75.9%), intranasal cocaine (47.2%), marijuana (51.5%), inhalants (26.5%), LSD (13.0%), and ecstasy (12.7%) in the previous 30 days.

Participants reported several risk behaviors, such as binge drinking in the previous 30 days (88.5%), irregular condom use (never or sometimes) with casual partners (64.0%), sex under the influence of alcohol (78.8%), sex under the influence of illicit drugs (73.0%), and sex with injecting drug users (7.4%).

The prevalence of transactional sex in the previous 12 months was estimated at 22.8% (95% CI: 18.6–27.7%). Prevalence in women and men was 52.7% (95% CI: 39.8–65.3) and 16.8% (95% CI: 12.8–21.7%), respectively, a significant difference (*p* < 0.001).

Table 1 shows the factors associated with the outcome in men. In multivariable analysis, crack use (adjusted prevalence ratio [APR]: 1.10; *p* = 0.009) and STI history (APR: 1.15; *p* = 0.040) remained as independent risk factors associated with transactional sex in this group.

The following remained factors associated with transactional sex in women, after multivariable analysis: history of homelessness (APR: 1.18; *p* = 0.041), crack use (APR: 1.30; *p* = 0.031), sex under the influence of drugs (APR: 1.29; *p* = 0.039), and sexual violence (APR: 1.22; *p* = 0.022) (Table 2).

## 4. Discussion

This study investigated the prevalence of transactional sex among NIDUs institutionalized in Goiás, Central Brazil. To the best of our knowledge, this is the first study to investigate the risk factors for this behavior in a sample of NIDUs in Brazil, a population highly vulnerable to HIV infection. Furthermore, this investigation addresses an important gap in the literature, assessing the factors associated with transactional sex NIDUs, stratified by sex. The results reveal a high prevalence of transactional sex in the individuals investigated.

The prevalence of transactional sex observed in this study (22.8%) was similar to that estimated in other studies on illicit drug users in different geographical locations. In San Salvador (El Salvador), Dickson-Gomez et al. [5] found a prevalence of 18.5% of exchanging sex for drugs and 13.5% of exchanging sex for crack, in a study carried out with 420 noninstitutionalized crack users. In São Paulo (Southeast Region), a study conducted out with 350 crack users under treatment for addiction estimated a rate of 14.0% [11]. In Baltimore, Maryland (USA), research conducted with 543 noninstitutionalized drug users found a transactional sex prevalence of 12.3% [12]. In Rio de Janeiro (Southeast Region) and Salvador (Northeast Region), an investigation carried out with 159 noninstitutionalized crack users found a transactional sex rate of 11.9% [13]. Another study conducted in Rio de Janeiro with 111 crack users under treatment found a transactional sex rate of 15.3% [14]. In Goiânia (Midwest Region), Guimarães et al. [4] estimated a prevalence of 18.9% in a study of 588 institutionalized crack users.

The prevalence of transactional sex differs between men and women, including their behavioral and social determinants [15]. Studies conducted in populations of illicit drug users have shown that women have higher transactional sex rates than men [6, 12, 13]. For example, a study conducted with a sample of drug users under treatment in the USA also found a significantly higher prevalence of transactional sex in women than in men in the previous year (41.4% versus 11.2%; *p* < 0.001) [6]. In Baltimore (USA), the frequency of transactional sex in the previous six months was greater in women than in men in a sample of illicit drug users (25.6% versus 9%, *p* < 0.001) [12]. In Rio de Janeiro and Salvador, research conducted with crack users showed a 19-fold higher prevalence of transactional sex in women than in men (45.7% versus 2.4%; *p* < 0.001) [13].

Indeed, the prevalence of transactional sex was statistically higher in women, confirming their vulnerability regarding this behavior. In addition to biological vulnerability, women who use drugs exhibit multiple determinants that increase their risk of HIV infection, including STI history, intimate partner sexual violence, low access to health services, and, in particular, transactional sex [16]. For example, a study conducted with female crack users in Brazil showed that the number of cases of HIV infection is associated with behaviors such as the exchange of sex for money and/or drugs [17]. Interventions and HIV infection control policies among NIDUs must consider the greater risk of women adopting this behavior and increase preventive measures for females.

The results of this study indicate that homelessness is a transactional sex predictor in women. This finding suggests that economic factors, in addition to the craving for drugs, are crucial in transactional sex [7].

Crack use is one of the major contributors to HIV infection epidemics in some locations, such as Brazil and the Caribbean [1, 18]. This drug is highly addictive, causing rapid and intense effects. Craving, defined as the uncontrollable urge to use drugs, causes individuals to engage in risk behaviors (e.g., high number of sexual partners, unprotected sex, and transactional sex), which may explain the high prevalence of this behavior in these individuals [19, 20]. In addition, crack users exhibit higher rates of transactional sex than users of other psychoactive substances [1]. As reported by other authors [3, 6], we observed an association between crack use and transactional sex in men and women, confirming the risk of this subgroup of users for this behavior.

In this study, three risk markers were associated with transactional sex: STI history in men and sex under the influence of illicit drugs and sexual violence in women. Illicit drug users who engage in transactional sex are less likely to negotiate condom use and are more at risk for unprotected sex and other behaviors for STI [1, 6, 21].

STI history has been used as an indirect marker of risk behaviors, such as transactional sex, among illicit drug users undergoing treatment [22]. Sexual violence is associated with behavioral changes such as increased risk behaviors (e.g., inconsistent condom use and anal sex) and HIV infection epidemics around the world, especially in women [23]. In addition, transactional sex also increases the chance of sexual violence due to differences in the power dynamics of the actors involved [24]. Given that sexual violence may have occurred in the context of transactional sex activities [9], this association should be interpreted with caution. In this respect, the relationship between transactional sex and risk behaviors suggests a complex association between factors, raising the risk of HIV infection in NIDUs.

This study must be interpreted in the context of its limitations. Its cross-sectional design does not allow establishing a temporal and causal relationship between transactional sex and risk factors [6]. This analysis only includes institutionalized individuals and cannot be generalized to other populations of drug users. The transactional sex variable was limited since it was not possible to assess the frequency of this behavior and we excluded nonconsensual transactional sex. Data were self-reported and subject to recall bias and social desirability of response biases and may be under- or overestimated. Despite its limitations, this study showed high prevalence of transactional sex in the NIDUs investigated, confirming the vulnerability of these individuals to HIV infection.

## 5. Conclusion

In conclusion, the high rates of transactional sex observed in NIDUs may play a key role in HIV transmission among NIDUs, in their social networks and the general population. Crack use and STI history were risk factors for transactional sex in men. A history of homelessness, crack use, sex under the influence of drugs, and history of sexual violence were risk factors for transactional sex in women.

Preventive actions should be intensified in NIDUs involved in transactional sex, especially for the most vulnerable subgroups (women, individuals with a history of homelessness, crack users, individuals with a history of violence, and STI). This includes programs and policies aimed at reducing sexual risk, such as regular testing for HIV and other infections, access to condoms, promotion of safe sex practices, and coordination between drug rehabilitation treatment services and centers for STI testing and counseling. In addition, further studies should be conducted to assess the impact on and role of transactional sex in the HIV infection epidemic among NIDUs.

## Figures and Tables

**Table 1:** Prevalence and risk factors for transactional sex among male noninjecting illicit drug users.

Variables	Transactional sex^a^		Crude PR^d^ (95% CI)^e^	*p*	Adjusted^f^ PR^d^ (95% CI)^e^	*p*
	Pos.^b^/total^c^	%				
Age (years)			0.99 (0.99–1.00)	0.072	0.99 (0.99–1.00)	0.052
History of homelessness^g^						
No	37/223	16.6	1.00			
Yes	8/45	17.8	1.01 (0.91–1.12)	0.849		
Marijuana use^h^						
No	26/143	18.2	1.00			
Yes	19/125	15.2	0.97 (0.90–1.05)	0.513		
Intranasal use^h^						
No	27/148	18.2	1.00			
Yes	18/120	15.0	0.97 (0.90–1.05)	0.447		
Crack use^h^						
No	4/68	5.9	1.00		1.00	
Yes	41/200	20.5	1.13 (1.06–1.22)	<0.001	1.10 (1.02–1.18)	0.009
Noninjecting heroin use^h^						
No	42/258	16.3	1.00			
Yes	3/10	30.0	1.01 (0.83–1.24)	0.864		
Inhalant use^h^						
No	29/198	14.6	1.00			
Yes	16/70	22.9	1.07 (0.97–1.17)	0.136		
LSD use^h^						
No	36/231	15.6	1.00			
Yes	9/37	24.3	1.07 (0.95–1.21)	0.228		
Ecstasy use^h^						
No	40/233	17.2	1.00			
Yes	5/35	14.3	0.97 (0.87–1.08)	0.656		
Tobacco use						
No	1/20	5.0	1.00		1.00	
Yes	44/246	17.9	1.12 (1.01–1.24)	0.023	1.07 (0.96–1.19)	0.170
Binge drinking^h^						
No	7/30	23.3	1.00			
Yes	38/238	16.0	0.94 (0.82–1.07)	0.351		
Condom use with casual partners^a^						
Always	26/72	22.2	1.00			
Never or sometimes	24/109	22.0	0.99 (0.90–1.10)	0.974		
Sex under the influence of alcohol						
No	10/55	18.2	1.00			
Yes	34/212	16.0	0.98 (0.89–1.08)	0.710		
Sex under the influence of drugs^a^						
No	10/74	13.5	1.00			
Yes	35/188	18.6	1.04 (0.96–1.13)	0.301		
Sex with injecting drug users^a^						
No	40/252	15.9	1.00			
Yes	5/16	31.5	1.13 (0.94–1.35)	0.163		
Sexual violence^a^						
No	41/255	16.1	1.00			
Yes	4/13	30.8	1.12 (0.92–1.37)	0.234		
STI history^a^						
No	28/197	14.2	1.00		1.00	
Yes	17/71	23.9	1.08 (0.99–1.18)	0.078	1.10 (1.01–1.21)	0.044

^a^In the previous 12 months; ^b^positive; ^c^the denominator reflects the number of valid responses; ^d^prevalence ratio; ^e^95% confidence interval; ^f^adjusted for age, crack use, tobacco use, and STI history; ^g^in the previous six months; ^h^in the previous 30 days.

**Table 2:** Prevalence and risk factors for transactional sex among female noninjecting illicit drug users.

Variables	Transactional sex^a^		Crude PR^d^ (95% CI)^e^	*p*	Adjusted^f^ PR^d^ (95% CI)^e^	*p*
	Pos.^b^/total^c^	%				
Age (years)			0.99 (0.99–1.00)	0.383	1.00 (0.99–1.01)	0.621
History of homelessness^g^						
No	19/42	45.2	1.00		1.00	
Yes	10/13	76.9	1.21 (1.03–1.54)	0.034	1.18 (1.01–1.38)	0.041
Marijuana use^h^						
No	4/14	28.6	1.00		1.00	
Yes	25/41	61.0	1.25 (1.01–1.54)	0.034	1.09 (0.85–1.41)	0.462
Intranasal cocaine use^h^						
No	10/23	43.5	1.00			
Yes	19/32	59.4	1.11 (0.92–1.32)	0.249		
Crack use^h^						
No	2/10	20.0	1.00		1.00	
Yes	27/45	60.0	1.33 (1.06–1.67)	0.013	1.30 (1.04–1.64)	0.021
Inhalant use^h^						
No	19/40	47.5	1.00			
Yes	10/15	66.7	1.12 (0.94–1.35)	0.181		
LSD use^h^						
No	27/50	54.0	1.00			
Yes	2/5	40.0	0.90 (0.65–1.25)	0.562		
Ecstasy use^h^						
No	25/49	51.0	1.00			
Yes	4/6	66.7	1.10 (0.86–1.41)	0.434		
Tobacco use^h^						
No	2/3	66.7	1.00			
Yes	27/52	51.9	0.91 (0.65–1.27)	0.588		
Binge drinking^h^						
No	3/7	42.9	1.00			
Yes	36/48	54.2	1.07 (0.81–1.42)	0.587		
Condom use with casual partners^a^						
Always	4/5	80.0	1.00			
Never or sometimes	19/28	67.9	0.93 (0.74–1.16)	0.541		
Sex under the influence of alcohol^a^						
No	5/13	38.5	1.00			
Yes	24/41	58.5	1.14 (0.92–1.42)	0.218		
Sex under the influence of drugs^a^						
No	2/11	18.2	1.00		1.00	
Yes	26/42	61.9	1.36 (1.10–1.69)	0.004	1.29 (1.01–1.65)	0.039
Sex with injecting drug users^a^						
No	25/47	53.2	1.00			
Yes	4/8	50.0	0.97 (0.76–1.25)	0.870		
Sexual violence^a^						
No	10/29	34.5	1.00		1.00	
Yes	18/25	72.0	1.27 (1.08–1.50)	0.004	1.22 (1.03–1.45)	0.022
STI history^a^						
No	22/44	50.0	1.00			
Yes	7/11	63.6	1.09 (0.89–1.33)	0.397		

^a^In the previous 12 months; ^b^positive; ^c^the denominator reflects the number of valid responses; ^d^prevalence ratio; ^e^95% confidence interval; ^f^adjusted for age, history of homelessness, marijuana use, crack use, sex under the influence of illicit drugs, and sexual violence; ^g^in the previous six months; ^h^in the previous 30 days.

## References

[1] Strathdee S. A., Stockman J. K. (2010). Epidemiology of HIV among injecting and non-injecting drug users: current trends and implications for interventions. *Current HIV/AIDS Reports*.

[2] Bobashev G. V., Zule W. A., Osilla K. C., Kline T. L., Wechsberg W. M. (2009). Transactional sex among men and women in the south at high risk for HIV and other STIs. *Journal of Urban Health*.

[3] Duff P., Tyndall M., Buxton J., Zhang R., Kerr T., Shannon K. (2013). Sex-for-Crack exchanges: associations with risky sexual and drug use niches in an urban Canadian city. *Harm Reduction Journal*.

[4] Guimarães R. A., da Silva L. N., França D. D. D. S., Del-Rios N. H. A., Carneiro M. A. D. S., Teles S. A. (2015). Risk behaviors for sexually transmitted diseases among crack users. *Revista Latino-Americana de Enfermagem*.

[5] Dickson-Gomez J., Lechuga J., Glasman L., Pinkerton S., Bodnar G., Klein P. (2013). Prevalence and incidence of HIV and sexual risk behaviors in crack users in the San Salvador Metropolitan Area, El Salvador. *World Journal of AIDS*.

[6] Burnette M. L., Lucas E., Ilgen M., Frayne S. M., Mayo J., Weitlauf J. C. (2008). Prevalence and health correlates of prostitution among patients entering treatment for substance use disorders. *Archives of General Psychiatry*.

[7] Newman P. A., Rhodes F., Weiss R. E. (2004). Correlates of sex trading among drug-using men who have sex with men. *American Journal of Public Health*.

[8] World Health Organization http://www.who.int/substance_abuse/terminology/ICD10ClinicalDiagnosis.pdf?ua=1.

[9] Okigbo C. C., McCarraher D. R., Chen M., Pack A. (2014). Risk factors for transactional sex among young females in post-conflict Liberia. *African Journal of Reproductive Health*.

[10] Coutinho L. M. S., Scazufca M., Menezes P. R. (2008). Methods for estimating prevalence ratios in cross-sectional studies. *Revista de Saude Publica*.

[11] de Carvalho H. B., Seibel S. D. (2009). Crack cocaine use and its relationship with violence and HIV. *Clinics*.

[12] Plitt S. S., Sherman S. G., Strathdee S. A., Taha T. E. (2005). Herpes simplex virus 2 and syphilis among young drug users in Baltimore, Maryland. *Sexually Transmitted Infections*.

[13] Bertoni N., Burnett C., Cruz M. S. (2014). Exploring sex differences in drug use, health and service use characteristics among young urban crack users in Brazil. *International Journal for Equity in Health*.

[14] Cruz M., Bertoni N., Bastos F. I., Burnett C., Gooch J., Fischer B. (2014). Comparing key characteristics of young adult crack users in and out-of-treatment in Rio de Janeiro, Brazil. *Substance Abuse Treatment, Prevention, and Policy*.

[15] Ahrens K. R., Katon W., McCarty C., Richardson L. P., Courtney M. E. (2012). Association between childhood sexual abuse and transactional sex in youth aging out of foster care. *Child Abuse and Neglect*.

[16] Campbell J. C., Lucea M. B., Stockman J. K., Draughon J. E. (2013). Forced sex and HIV risk in violent relationships. *American Journal of Reproductive Immunology*.

[17] Nappo S. A., Sanchez Z., De Oliveira L. G. (2011). Crack, AIDS, and women in São Paulo, Brazil. *Substance Use & Misuse*.

[18] Hacker M. A., Malta M., Enriquez M., Bastos F. I. (2005). Human immunodeficiency virus, AIDS, and drug consumption in South America and the Caribbean: epidemiological evidence and initiatives to curb the epidemic. *Revista Panamericana de Salud Publica*.

[19] Atkinson J. S., Williams M. L., Timpson S. C., Schönnesson L. N. (2010). Multiple sexual partnerships in a sample of African-American crack smokers. *AIDS and Behavior*.

[20] de Sá L. C., de Araújo T. M. E., Griep R. H., Campelo V., Monteiro C. F. D. S. (2013). Seroprevalence of Hepatitis C and factors associated with this in crack users. *Revista Latino-Americana de Enfermagem*.

[21] Strathdee S. A., Philbin M. M., Semple S. J. (2008). Correlates of injection drug use among female sex workers in two Mexico-U.S. border cities. *Drug and Alcohol Dependence*.

[22] Guimarães R. A., Lucchese R., De Felipe R. L. (2015). Factors associated with the report of sexually transmitted infections in drug users, Central Brazil: a cross-sectional study. *Sexually Transmitted Infections*.

[23] Azim T., Bontell I., Strathdee S. A. (2015). Women, drugs and HIV. *International Journal of Drug Policy*.

[24] Klot J. F., Auerbach J. D., Berry M. R. (2013). Sexual violence and HIV transmission: summary proceedings of a scientific research planning meeting. *American Journal of Reproductive Immunology*.

